# Diagnostic accuracy using low-dose *versus* standard radiation dose CT in suspected acute appendicitis: prospective cohort study

**DOI:** 10.1093/bjs/znab383

**Published:** 2021-11-11

**Authors:** Jussi Haijanen, Suvi Sippola, Ville Tammilehto, Juha Grönroos, Siiri Mäntyoja, Eliisa Löyttyniemi, Hannele Niiniviita, Paulina Salminen

**Affiliations:** 1 Division of Digestive Surgery and Urology, Turku University Hospital, Turku, Finland; 2 Department of Surgery, University of Turku, Turku, Finland; 3 Department of Surgery, Jyväskylä Central Hospital, Jyväskylä, Finland; 4 Department of Radiology, Medical Imaging Centre of Southwest Finland, Turku University Hospital, Turku, Finland; 5 Department of Biostatistics, University of Turku, Turku, Finland; 6 Department of Medical Physics, Turku University Hospital, Turku, Finland

## Abstract

**Background:**

Contrast-enhanced CT is the reference standard used in diagnostic imaging for acute appendicitis in adults. The radiation dose has been of concern. This study aimed to assess whether a lower radiation dose would affect the diagnostic accuracy of CT.

**Methods:**

This was a prospective single-centre cohort study of patients (aged over 16 years) with suspected appendicitis evaluated for enrolment in concurrent APPAC II–III trials. The diagnostic accuracy of contrast-enhanced low- and standard-dose CT was compared with study protocols guiding imaging based on BMI; this enabled direct CT imaging comparison only in patients with a BMI below 30 kg/m^2^. The on-call CT diagnosis was compared with the final clinical diagnosis.

**Results:**

Among all 856 patients investigated, the accuracy of low-dose (454 patients) and standard-dose (402 patients) CT in identifying patients with and without appendicitis was 98·0 and 98·5 per cent respectively. In patients with a BMI under 30 kg/m^2^, respective values were 98·2 per cent (434 patients) and 98·6 per cent (210 patients) (*P* = 1·000). The corresponding accuracy for differentiating between uncomplicated and complicated acute appendicitis was 90·3 and 87·6 per cent in all patients, and 89·8 and 88·4 per cent respectively among those with a BMI below 30 kg/m^2^ (*P* = 0·663). The median radiation dose in the whole low- and standard-dose CT groups was 3 and 7 mSv respectively. In the group with BMI below 30 kg/m^2^, corresponding median doses were 3 and 5 mSv (*P* < 0·001).

**Conclusion:**

Low- and standard-dose CT were accurate both in identifying appendicitis and in differentiating between uncomplicated and complicated acute appendicitis. Low-dose CT was associated with a significant radiation dose reduction, suggesting that it should be standard clinical practice at least in patients with a BMI below 30 kg/m^2^.

## Teaser

In this prospective cohort study, low- and standard-dose CT were accurate both in identifying appendicitis and in differentiating between uncomplicated and complicated acute appendicitis. Low-dose CT was associated with a significant radiation dose reduction, suggesting that it should be standard clinical practice at least in patients with a BMI under 30 kg/m^2^.

## Introduction

Increasing evidence for the safety, efficacy, and feasibility of non-operative treatment for uncomplicated acute appendicitis^1–6^ has set new demands for the accuracy of diagnostics in acute appendicitis. As emergency appendicectomy is no longer considered the only treatment alternative for patients with uncomplicated acute appendicitis^1^^,^^5–8^, the emphasis of appendicitis diagnostics has shifted from solely assessing whether the patient has appendicitis or not towards differentiating between uncomplicated and complicated acute appendicitis. Acute appendicitis presenting with perforation, tumour, gangrene, or periappendicular abscess has traditionally been considered as complicated acute appendicitis requiring emergency appendicectomy. An exclusion has been patients with a restricted periappendicular abscess that is often initially treated with antibiotics followed by interval appendicectomy. In addition, the presence of an appendicolith has been shown to be associated with a more complicated course of acute appendicitis, markedly increasing the risk of failure of non-operative treatment^9–14^. Based on recent evidence^14^, appendicolith should currently be considered a feature of complicated acute appendicitis.

In both the previous APPAC (APPendicitis ACuta) study^15^, which compared appendicectomy with antibiotic therapy, and the follow-up studies APPAC II^6^ and APPAC III^16^, which focused on optimizing non-operative treatment of uncomplicated acute appendicitis, an intraluminal appendicolith on initial CT was an exclusion criterion. The conclusive, standardized, and uniform definition of complicated acute appendicitis is under active research and discussion, especially in terms of diagnostic imaging findings needed for accurate preintervention diagnosis enabling the assessment of all available treatment options.

Imaging has become standard in the diagnosis of acute appendicitis, reducing both the negative appendicectomy rate and overall treatment costs^17^^,^^18^. With current substantial evidence and increasing interest in non-operative treatment for uncomplicated acute appendicitis^8^, high preinterventional sensitivity in identifying patients with complicated acute appendicitis is mandatory and this is best achieved with imaging^19^^,^^20^. Although ultrasonography is often considered the first-line imaging^21^ in children to avoid unwanted radiation, CT is the standard for diagnostic imaging for suspected acute appendicitis in adults. However, the radiation exposure associated with CT is an indisputable disadvantage, especially as the majority of patients with suspected acute appendicitis are young adults^22^^,^^23^. As the development of cancer is a multifactorial process comprising both numerous patient-related factors together with the potential burden of ionizing radiation, assessing the exact risk reduction following a certain CT dose reduction is very challenging^23^. However, as, according to the linear no-threshold model, even a small reduction in radiation dose reduces the risks of radiation in a large patient population, the principle of using as low a dose as reasonably achievable should be adhered to, with the aim of reducing the radiation dose whenever possible.

Although there is accumulating evidence showing equal accuracy for low- and standard-dose CT protocols in diagnosing acute appendicitis, with markedly decreased exposure to radiation in low-dose modalities^24–28^, the large-scale implementation of low-dose protocols in clinical practice has been slow. This may be due to a lack of comprehensive data on the accuracy of these low-dose protocols in assessing the severity of appendicitis. The OPTICAP study assessed this clinically important topic, first by optimizing the imaging *in vitro*^29^, followed by a prospective interpatient randomized study^30^; the latter enrolled 60 patients with a BMI under 30 kg/m^2^ and suspected acute appendicitis undergoing consecutive low- and standard-dose imaging. The low-dose CT was shown to be non-inferior to standard-dose CT in terms of accuracy both in diagnosing appendicitis and non-appendicitis, as well as in differentiating between uncomplicated and complicated acute appendicitis. A recent study^31^ conducted in an Asian population corroborated these results.

The aim of the present large single-centre prospective cohort study was to compare the accuracy of low- *versus* standard-dose CT in both diagnosing acute appendicitis, and in differentiating between uncomplicated and complicated acute appendicitis.

## Methods

### Design

This single-centre prospective cohort study was undertaken to compare the accuracy of low- and standard-dose CT in both diagnosing acute appendicitis, and in differentiating between complicated and uncomplicated acute appendicitis. Patients were enrolled in the study concurrently with assessing eligibility for the APPAC II^6^ and APPAC III^16^ RCTs, which enrolled adult patients with CT-confirmed uncomplicated acute appendicitis. The study followed the STARD reporting guidelines for diagnostic accuracy studies^32^.

### Ethics

The APPAC II and III study protocols were approved by the ethics committee at the Hospital District of Southwest Finland and by institutional research boards at each participating site. In accordance with the APPAC II and III study protocols^33^^,^^34^, patient data were recorded prospectively in an online database and signed informed consent was obtained from all patients evaluated for eligibility.

### Participants

Patients aged 16 years or older were enrolled for the present study between 4 April 2017 and 27 November 2018 at Turku University Hospital, Finland. During the enrolment period, all patients with a clinical suspicion of acute appendicitis admitted to the emergency room were included in this cohort study. Patients randomized to the concurrent APPAC II study received either oral antibiotic monotherapy or intravenous antibiotics followed by oral antibiotic treatment, whereas those in the APPAC III study received either treatment with antibiotics or with placebo. Patients excluded from these RCTs (owing to complicated appendicitis, no appendicitis, or refusal to participate) were treated according to the discretion of the surgeon on call. Furthermore, because of the acute-care surgery setting of this trial, to ensure thorough inclusion of all patients admitted to the emergency room with suspected acute appendicitis during the enrolment period, hospital records were checked retrospectively for the ICD-10 appendicitis diagnosis codes (K35·0, K35·1, K35·9), procedure codes for open and laparoscopic appendicectomy, and referrals to CT owing to suspected acute appendicitis.

### Test methods

In accordance with the APPAC II and III study protocols^16^^,^^33^, for all patients with suspected acute appendicitis, surgeons or acute-care physicians on call were instructed to perform diagnostic low-dose abdominal CT in patients with a BMI under 30 kg/m^2^ and standard-dose abdominal CT in those with a BMI of 30 kg/m^2^ or higher. Patients were imaged (Aquilion One^TM^; Toshiba Medical Systems, Otawara, Japan) from the diaphragm to the symphysis during the early portal venous phase with routine patient weight-adjusted intravenous iodinated contrast medium (1·5 ml/kg; concentration 350 mg iodine per ml; injection rate 3 ml/s). The standard-dose protocol used 120 kV, standard iterative reconstruction, and had a noise index of 12·5. The low-dose protocol used 100 kV, standard iterative reconstruction, and had a noise index of 14·5^29^^,^^30^. The dose length product (DLP) for each CT examination was registered. Additionally, the estimation of effective dose was calculated based on the individual DLP and the coefficients as described by Huda and colleagues^35^.

### Diagnostic assessment

#### Index test (CT)

The radiological criteria for acute appendicitis shown in *Table 1* were used in the OPTICAP trial^30^ as well as in the APPAC II^33^ and III^16^ trials. The experience level among the radiologists on call who assessed the CT findings varied from attending radiologist to trainees; all, however, had a minimum of 3 years of experience in radiology. According to the images evaluated by the radiologist on call, all patients were classified into one of the following three radiological diagnosis groups: uncomplicated acute appendicitis, complicated acute appendicitis, or normal appendix. Patients with an alternative disease diagnosis on CT and no pathological appendiceal findings were classified as having a normal appendix.

**Table 1:** Structured radiological report including radiological criteria and categorization of acute appendicitis

**Appendix visualization**
Report one of the following: not visualized, partly or unclearly visualized, completely visualized
**Appendix transverse diameter (mm)**
**Probability of appendicitis**
Report one of the following: not likely, rather unlikely, rather likely, very likely
**Categorization of appendicitis**
Report either I or II, if any:
I Uncomplicated appendicitis: transverse diameter > 6 mm with typical findings
Wall thickening and enhancement
Periappendiceal oedema and/or minor amount of fluid
II Complicated appendicitis: abovementioned criteria for appendicitis with at least one of the following:
Appendicolith: > 3 mm stone within appendix
Abscess: periappendiceal walled collection with enhancing walls
Perforation: appendiceal wall enhancement defect and periappendiceal excess of fluid and/or infectious phlegmon and/or extraluminal air
Tumour: tumour-like prominence of appendix
**Other diagnosis**
Report if any: diverticulitis, complicated ovarian cyst, pelvic inflammatory disease, colitis, ileitis, intestinal obstruction or ileus, ureter stone, hydronephrosis, tumour, other diagnosis

#### Reference standard

After undergoing treatment based either on participation in the ongoing trials or the standard clinical treatment, the patients were classified into one of three groups according to the final clinical diagnosis based on blinded assessment by three researchers using the following data: diagnostic CT, operative findings and histopathology (in those who had operative treatment), and patient recovery.

For patients undergoing appendicectomy, the finding of intramural neutrophil invasion of the removed appendix was required for the histopathological diagnosis of acute appendicitis. The surgical finding of perforation was evaluated as complicated acute appendicitis as well as gangrene of the appendix, if supported by both surgery and histopathology. Appendiceal neoplasm was classified as complicated acute appendicitis. In the event of discrepancy in the final clinical diagnosis between junior surgeon researchers, the final clinical diagnosis was made by the senior surgeon researcher. Patients presenting with an appendicolith either on CT or during operation were given the final clinical diagnosis of complicated acute appendicitis.

For patients treated without surgery (normal appendix or uncomplicated acute appendicitis on CT treated conservatively), the CT diagnosis was set as the final clinical diagnosis after a 30-day follow-up, if there was no readmission or any other contact with the hospital suggesting an alternative diagnosis. The final diagnosis for patients with a periappendicular abscess treated primarily with antibiotics followed by interval appendicectomy was set after the appendicectomy to ensure that the operative finding did not suggest an alternative diagnosis. If the appendix could not be visualized on CT, but another clear alternative diagnosis was suggested by the radiologist, this alternative diagnosis was determined as the final clinical diagnosis and the appendix was deemed normal. If the appendix could not be visualized, and no alternative diagnosis was suggested on CT (inconclusive CT), the final clinical diagnosis was based on the possible operative and histopathological findings. Patients with inconclusive CT findings who did not undergo surgery at any point were excluded as no reliable clinical diagnosis could be established.

### Assessment of accuracy of low- and standard-dose CT

The diagnosis suggested by the radiologist on call based on CT was compared with the final clinical diagnosis established by the investigators. All patients were included in the analyses when assessing the accuracy of the CT modalities in identifying patients with and without acute appendicitis. Only patients with appendicitis were included in the evaluation of the accuracy of differential diagnostics between uncomplicated and complicated acute appendicitis; those with a final clinical diagnosis of a normal appendix were excluded.

### Subgroups based on BMI and appendicolith status

To evaluate the effect of BMI on diagnostic accuracy, subgroup analyses were conducted, including patients with a BMI under 30 kg/m^2^ and those with an BMI of 30 kg/m^2^ or more. To evaluate the effect of possible appendicolith on the accuracy of low- and standard-dose CT, a subgroup analysis was conducted including only patients without an appendicolith identified on CT or during operation. As the presence and role of appendicolith has not yet been fully established, a subgroup analysis was undertaken including all patients, but the finding of appendicolith at imaging or surgery was disregarded altogether, and both the radiological diagnosis and the final clinical diagnosis were based on all other predefined criteria.

### Statistical analysis

Continuous variables are summarized as median (range or interquartile range). Age is presented as median (range), and categorical variables as numbers with percentages. As there were notable differences in BMI between low- and standard-dose CT groups because the study protocol guided the imaging based on patient BMI, formal statistical analyses were undertaken only for the subgroup of patients with a BMI under 30 kg/m^2^. For the whole study group, the descriptive data are presented without formal statistical analysis between the groups. Wilcoxon rank-sum test was used for analysis of radiation dose. For patients with a BMI under 30 kg/m^2^, accuracy comparison was performed with Fisher’s exact test and 95 per cent confidence intervals were calculated for accuracy. All tests were two-tailed and *P* < 0.050 was considered statistically significant. Analysis was done using SAS® 2for Windows® version 9·4 (SAS Institute, Cary, North Carolina, USA).

## Results

**Fig. 1:**
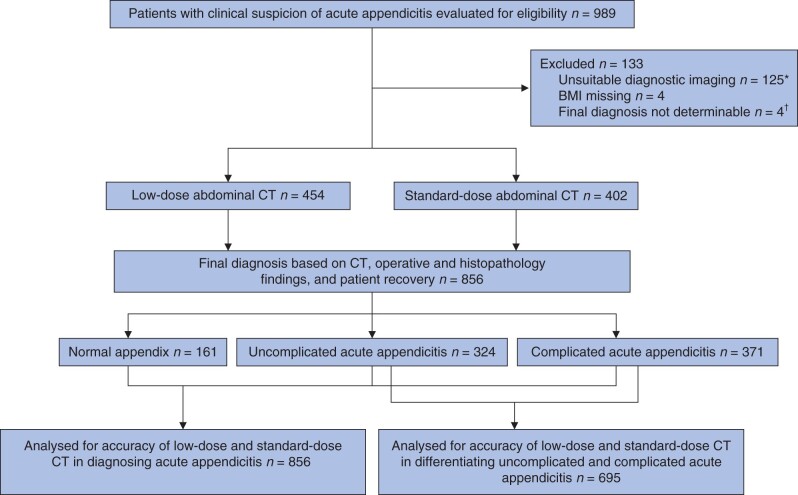
Study flow chart *The majority of these patients underwent ultrasound examination, non-contrast-enhanced CT, or MRI; some patients had surgery without diagnostic imaging. †Two patients were transferred from the emergency department to another hospital for treatment. In two other patients, the appendix could not be visualized on CT and no alternative pathology was found on CT; they did not undergo surgery and recovered with antibiotics, but the final clinical diagnosis could not be determined reliably.

A total of 989 patients were admitted to the emergency room with suspected acute appendicitis (*Fig.  1*). Among the 856 included patients, the median age was 37 (range 16–87) years and 52 per cent were female. Some 53⋅0 per cent (454 of 856) underwent low-dose CT and 47⋅0 per cent (402 of 856) standard-dose CT. Baseline demographics are summarized in *Table 2*.

**Table 2:** Baseline characteristics of patients included in analyses

	Low-dose abdominal CT (*n* = 454)	Standard-dose abdominal CT (*n* = 402)
**Sex ratio (F : M)**	244 : 210	204 : 198
Age (years)*	31 (16–75)	47 (16–87)
**BMI** (kg/m^2^)*	24·7 (16·6–42·7)	29·5 (16·9–56·8)
≥ 30	20 (4·4)	192 (47·8)
< 30	434 (95·6)	210 (52·2)
**C-reactive protein (mg/l)** *	35·0 (1·0–462·0)	42·5 (1·0–418·0)
**White blood cell count (×10^9^/l)** *	12·3 (2·7–27·3)	12·8 (2·9–30·9)

Values in parentheses are percentages unless indicated otherwise;

*values are median (range).

The overall accuracy of low- and standard-dose CT in identifying patients with and without acute appendicitis was 98·0 per cent (445 of 454) and 98·5 per cent (396 of 402) respectively, and the accuracy for differentiating between uncomplicated and complicated acute appendicitis was 90·3 per cent (307 of 340) and 87·6 per cent (311 of 355) respectively. The sensitivity of low- and standard-dose CT for complicated acute appendicitis was 89·6 per cent (146 of 163) and 84·6 per cent (176 of 208), and the specificity was 91·0 per cent (161 of 177) and 91·8 per cent (135 of 147), respectively. The diagnostic accuracy between the two modalities was comparable in subgroup analyses in which patients were classified according to BMI or appendicolith status (*Table 3*).

**Table 3:** Subgroup analyses for accuracy of intravenous contrast-enhanced low- and high-dose abdominal CT in diagnosing acute appendicitis, and in differentiating complicated and uncomplicated acute appendicitis

	Accuracy in diagnosing acute appendicitis and no acute appendicitis (%)	Accuracy in differentiating complicated and uncomplicated acute appendicitis (%)
**BMI < 30 kg/m^2^ (*n* = 644)**		
Low dose	98·2 (426 of 434)	89·8 (290 of 323)
Standard dose	98·6 (207 of 210)	88·4 (176 of 199)
**BMI ≥ 30 kg/m^2^ (*n* = 212)**		
Low dose	95 (19 of 20)	100 (17 of 17)
Standard dose	98·4 (189 of 192)	86·5 (135 of 156)
**Presence of appendicolith selectively disregarded (*n* = 856)** *		
Low dose	98·0 (445 of 454)	80·9 (275 of 340)
Standard dose	98·5 (396 of 402)	75·8 (269 of 355)
**No appendicolith (*n* = 602)**†		
Low dose	97·3 (322 of 331)	86·2 (187 of 217)
Standard dose	97·8 (265 of 271)	81·7 (183 of 224)

*For this subgroup, the possible finding of appendicolith on CT or during surgery was not automatically considered a finding of complicated acute appendicitis, and the diagnosis and subcategorization of acute appendicitis was based on all other findings on CT, surgery, and histopathology. †Including only patients with no appendicolith visible on CT or during operation.

In the subgroup analysis of patients with a BMI under 30 kg/m^2^, the overall accuracy of low- and standard-dose CT in identifying patients with and without acute appendicitis was 98·2 per cent (426 of 434; 95 per cent c.i. 96·9 to 99·4 per cent) and 98·6 per cent (207 of 210; 95 per cent c.i. 97·0 to 100 per cent) respectively (*P* = 1·000), and the accuracy for differentiating between uncomplicated and complicated acute appendicitis was 89·8 per cent (290 of 323; 95 per cent c.i. 86·5 to 93·1 per cent) and 88·4 per cent (176 of 199; 95 per cent c.i. 84⋅0 to 92⋅9 per cent) respectively (*P* = 0·663).

### Radiation exposure

Among all patients imaged with CT, the median radiation dose was lower in the low-dose compared with the standard-dose group (3 and 7 mSv respectively). In the subgroup analysis including only patients with a BMI under 30 kg/m^2^, the median radiation dose was significantly lower in the low-dose than in the standard-dose group: 3 (i.q.r. 3–4) and 5 (4–7) mSv respectively (*P* < 0·001).

For 3 of 454 patients (0·7 per cent) in the low-dose group, the appendix was reported as normal on CT with a final diagnosis of acute appendicitis (2 uncomplicated and 1 complicated). No patient in the standard-dose group with a normal appendix on CT eventually had appendicitis.

### Alternative diagnoses

In 5 of 989 patients (4 who had low-dose imaging, and 1 who initially underwent low-dose CT followed by standard-dose CT 2 days later), the appendix could not be visualized by the radiologist and no alternative diagnosis was found, leaving the initial CT inconclusive. Two of these patients underwent exploratory laparoscopy and appendicectomy, with the finding of a normal appendix both at surgery and histopathology and no alternative diagnosis at laparoscopy. Three of the five patients were treated successfully with antibiotics and one patient was diagnosed with pelvic inflammatory disease during the primary hospital admission; no final clinical diagnosis could reliably be determined for the remaining two patients and they were excluded from the analyses.

### Role of appendicolith

When patients with an intraluminal appendicolith visible on CT or at surgery were classified as having complicated acute appendicitis, 371 of 695 patients (53·4 per cent) with appendicitis were considered to have complicated appendicitis. If the presence of an appendicolith was disregarded and complicated acute appendicitis was diagnosed based on the finding of perforation, gangrene, tumour, or abscess, 242 patients (34·8 per cent of all those with appendicitis) were classified as having complicated acute appendicitis.

## Discussion

In this study, which reflects routine clinical practice in acute-care surgery, low- and standard-dose abdominal CT were equally accurate in both diagnosing acute appendicitis and in differentiating between uncomplicated and complicated acute appendicitis, corroborating existing evidence^24^^,^^25^^,^^28^^,^^30^^,^^31^. In addition, the median radiation dose associated with low-dose CT was lower than that used in standard-dose imaging. Increasing evidence for the safety, efficacy, feasibility^1–6^^,^^8^^,^^36^, and cost benefits^37^^,^^38^ of non-operative management of CT-confirmed uncomplicated acute appendicitis are gradually transitioning to clinical practice. Correspondingly, and concurrently with the changing treatment paradigms, the present findings of low-dose CT having similar accuracy with a markedly lower radiation dose should further encourage active implementation of low-dose modalities in acute appendicitis imaging, regardless of whether CT is used as first-line imaging or after, for example, an inconclusive ultrasound examination.

The recent APPAC II trial^6^ compared oral antibiotic monotherapy with intravenous followed by oral antibiotics for uncomplicated acute appendicitis with low-dose CT as the protocol diagnostic imaging for patients with a BMI below 30 kg/m^2^. In that trial, 207 (70·2 per cent) of the 295 patients treated with oral antibiotics only did not undergo appendicectomy within the first year, corroborating earlier results on the feasibility of antibiotic treatment of CT-confirmed uncomplicated acute appendicitis. Together with earlier promising results on the outpatient management of acute appendicitis^39^, the findings of the APPAC II trial encourage further pursuit of the possibilities for outpatient management in the future. With the challenges and burden imposed on the healthcare system by the COVID-19 pandemic, these findings highlight the importance of accurate preinterventional assessment of the severity of appendicitis. Accuracy in differentiating between uncomplicated and complicated acute appendicitis will enable both assessment of all treatment alternatives available to the patient, and possibly also the option to avoid admission to hospital altogether for some patients. The present results further support the findings of the APPAC II^6^ and OPTICAP^30^ trials that this preintervention assessment can be carried out in actual clinical practice using low-dose CT without compromising diagnostic accuracy.

Defining complicated acute appendicitis is complex, and the lack of standardized and uniform criteria for complicated acute appendicitis makes the comparison of studies challenging^27^. In a meta-analysis, Kim and colleagues^40^ reported 10 CT findings associated with complicated acute appendicitis that reached a pooled sensitivity of 92 per cent at the expense of specificity (43 per cent)^41^. In the present study, the sensitivity of low- and standard-dose CT was 89·6 and 84·6 per cent respectively, with a specificity of 91·0 and 91·8 per cent. When considering patients for potential non-operative treatment, the preferred diagnostic approach should aim to minimize the rate of false-negative results for complicated acute appendicitis. Accepting a higher false-positive rate, resulting only in some potentially unnecessary appendicectomies for uncomplicated acute appendicitis, is acceptable, with appendicectomy being a good treatment option in contrast to missing a diagnosis of complicated acute appendicitis. Future focus should be on seeking even more precise preinterventional diagnostic tools, first by identifying the most useful CT findings and potentially combining these with clinical features. For instance, Atema and co-workers^19^ reported a promising negative predictive value of 94·7 per cent for complicated appendicitis using a scoring system combining CT and clinical features.

The large pragmatic trial by the CODA collaborative^14^ corroborated previous findings that the presence of an appendicolith on CT is associated with a more complicated course of appendicitis, resulting in increased risk of treatment failure and complications if treated without surgery^9–13^. This further underlines the importance of accurate patient selection, with exclusion of patients presenting with an appendicolith from non-operative treatment evaluation as in the APPAC II and III trials^6^^,^^16^. The similar diagnostic accuracy of low- and standard-dose CT, regardless of appendicolith status, reported in this study enables this in clinical practice.

This study has certain limitations. Imaging was mainly performed according to the APPAC II^33^ and III^16^ study protocols, guiding patients with a BMI under 30 kg/m^2^ to low-dose CT and those with a higher BMI to standard-dose imaging^29^^,^^30^. Presumably owing to the study protocol-guided imaging, the patient groups for the two imaging modalities differed in terms of BMI, but also in terms of age and C-reactive protein level. To at least partially address the effects of these differences on the diagnostic accuracy, a subgroup analysis including only the 644 patients with a BMI under 30 kg/m^2^ was undertaken, which showed comparable accuracy of low- and standard-dose imaging modalities, similar to the main analysis. This subgroup analysis, which allowed comparison of the accuracy of the two CT modalities in a large prospective patient cohort, was feasible only because 210 patients with a BMI under 30 kg/m^2^ underwent standard-dose CT instead of low-dose CT as instructed by the ongoing trials; this reflects the challenges of a study carried out in a real-life acute-care setting. However, this study was still underpowered to assess the accuracy of low-dose CT in patients with a higher BMI (over 30 kg/m^2^), although higher BMI may not substantially decrease the accuracy in detecting acute appendicitis using low-dose CT^42–44^. As the prevalence of obesity is also ever increasing globally among young adults^45^, lowering the radiation exposure in obese patients is very important, and the effect of obesity on the accuracy of low-dose CT needs to be assessed further in future studies.

The higher median BMI of the standard-dose group limited the comparability of radiation dose between the groups as higher BMI is independently associated with an increase in radiation dose^46^. However, the subgroup analysis including only patients with a BMI under 30 kg/m^2^ showed a statistically significant decrease in the radiation dose among patients who underwent low-dose CT. This finding corroborated the results of the OPTICAP study^30^ in which the same patient was imaged using both low- and standard-dose protocols identical to the ones used here. The final diagnosis of 141 patients treated with antibiotics could be considered as being subject to verification bias without a histopathological reference standard. However, this risk of bias is likely to be minor as follow-up of the patients’ recovery extended to 30 days.

Even though the patients were registered prospectively, owing to the acute-care surgery setting, prospective data were supplemented retrospectively to ensure the inclusion of all patients with suspected acute appendicitis during the enrolment period. As no retrospective changes were made to the actual on-call interpretations of CT findings, this rechecking of hospital records enabled analysis of a comprehensive cohort of patients with appendicitis, which can be considered a strength of the study rather than a limitation.

Another strong element of the study is the acute-care surgery setting representative of actual clinical care, markedly adding to the reproducibility and generalizability of the present results in everyday practice. In the assessment of severity of appendicitis, the investigators did not rely solely on clinical surgical assessment or histopathology alone in determining the final clinical diagnosis as there may often be a discrepancy between the two^47^. The thorough assessment by three investigators, taking the surgical and histopathological findings as well as imaging, laboratory tests, and patient recovery into consideration, is a major strength of this study. Finally, to the authors’ knowledge, this is the most extensive study conducted to date assessing the diagnostic accuracy of low-dose CT in acute appendicitis in a Western population, supporting the earlier evidence reported in trials^24^^,^^28^ carried out mainly in Asian populations.

## Supplementary Material

znab383_Supplementary_Data
